# To evaluate rising caesarean section rate and factors contributing to it by using Modified Robson’s Criteria at a tertiary care hospital

**DOI:** 10.12669/pjms.38.7.5983

**Published:** 2022

**Authors:** Erum Majid, Shazia Kulsoom, Sara Fatima, Bader Faiyaz Zuberi

**Affiliations:** 1 Erum Majid, FCPS. Department of Obstetrics and Gynaecology, Ward-8, Jinnah Postgraduate medical centre, Karachi, Pakistan; 2 Shazia Kulsoom, FCPS. Department of Neurology, National Institute of Child Health Karachi, Pakistan. Department of Obstetrics and Gynaecology, Ward-8, Jinnah Postgraduate medical centre, Karachi, Pakistan; 3 Sara Fatima, FCPS. Department of Obstetrics and Gynaecology, Ward-8, Jinnah Postgraduate medical centre, Karachi, Pakistan; 4 Bader Faiyaz Zuberi, FPCS. Department of Medicine/Gastroenterology, Dow University of Health Sciences, Karachi, Pakistan

**Keywords:** Caesarean Section, Modified Robson’s Criteria, Placenta Accreta

## Abstract

**Objectives:**

To determine the frequency of caesarean section with its indication by grouping according to Modified Robson’s Criteria at JPMC.

**Methods:**

This is a retrospective study done from 1st January to 30th June 2018. Records of all Caesarean Section performed during the study period were retrieved from elective and emergency operation theatres. Data was extracted from the emergency and elective Theatres registers. and entered in study proforma. Patients with missing data were contacted via a given phone number on file and data collected. None of the Patients Data was totally missing ,as all entries made were done very carefully .Data was entered in SPSS version 26.0. Group-11 and 12 were added in order to identify the main reason for the increase in caesarean sections.

**Results:**

Total number of deliveries in six months were 3400. Our study showed a Caesarean Section rate of 36.5 per all live births. The major group contributing was Group-5 (56%). Foetal distress (Group-12) and Primigravida with Inductions or caesarean section before labour (Group-2) showed nearly the same percentages 13.5% and 14% respectively.

**Conclusions:**

Planning of the caesarean section of primigravida should be carefully decided. The role of safe VBAC plays the key role in decreasing Cesarean Section rate. Moreover, foetal distress and caesareans in Primigravida should be evaluated with great accuracy to decrease the caesarean section rate.

**LIST OF ABBREVIATIONS**
CS: Caesarean Section,
VBAC: Vaginal Birth After Caesarean,
SD: Standard Deviation,
Χ^2^: Chi-Square.

## INTRODUCTION

Rising Caesarean Section (CS) rates are a major public health concern all over the world.^1^ Due to this drastic change in the new era, the complex surgical difficulties faced by an obstetrician has become a dilemma.^2^ The overwhelming work at tertiary care centres is making the trainees and obstetricians’ life strenuous. The leap seen in placenta accreta spectrum (PAS), rupture uterus and obstetrical hysterectomies has raised maternal morbidity and mortality. The financial expenses of the procedures have certainly impoverished the poor people.

If we look a decade back, the rate and complication caused by CS were minor. Comparing the PAS rate of Placenta Accreta in 1970 was one in 2570 which rose to one 1 in 272 in 2016.^1^,^3^ Patients with PAS require hysterectomy and a longer hospital stay.^4^ Today the main cause of PAS is uterine scar. This rise may be due to lack of expertise, shortage of time, patients’ intolerant attitude, improper foetal assessment, and an over-conscious patient.

Auditing helps to reduce CS rate by increasing awareness among doctors. Robson’s Ten Group Classification System (TGCS) was introduced in 2001 for classifying CS.^5^ (TGCS) is used internationally to classify caesarean section. TGCF can be modified according to the type of patients, hospital facilities and outcome. We reclassified TGCF into Group-11 and Group-12 due to its limitations. Group-11 was classified for the Placenta Previa and Accreta Spectrum. Foetal distress, which was one of the major causes of caesarean, was categorized as Group-12. Both these new subgroups were added as they contributed largely to caesarean section rate. Moreover, it was difficult to fit these subgroups in Robson’s Ten groups.

We tried to focus and figure out the main reason for the rising caesarean section. As JPMC caters a large area of population, keeping a vigilant insight in the causes of caesarean section is necessary. The main idea behind the study was to set our sights on what the reasons were for performing CS and create awareness to reduce the rising CS rate.

## METHODS

This is a retrospective study done from 1^st^ January to 30^th^ June 2018 at Jinnah Postgraduate Medical Centre, Karachi. Ethical approval was taken from institutional review board (IRB No 37632) at JPMC. Informed consent was not required and data were retrieved from ward, emergency and elective operation theatre registers.. Detailed history regarding the cause of caesarean section, maternal characteristics (age, parity and gravidity, medical or surgical past history), past obstetric history, pregnancy related information (Spontaneous or induced labour, number of foetuses, foetal presentation) and maternal and foetal (foetal weight, Apgar score, and any foetal anomaly) outcome were retrieved. After extracting data from files and registers, grouping was done as per Modified Robsons Criteria. The caesarean section register is maintained with entries soon after doing the caesarean section in both elective and emergency operation theatre. Total no of deliveries were extracted from daily stats entered on computer.

### Data Analysis

Data was collected for maternal age, parity, gestational age, parity, maternal and foetal outcome and weight of new-born. Qualitative data like parity foetal outcome and weight of new-born were presented in frequencies and percentages. Quantitative data like maternal age, gestational age- were presented in means ±SD. The overall CS rate and each group’s contribution were calculated. Frequencies of age groups, gestational age, procedure done and weight of outcome were compared according to gravida parity with Χ^2^-test.

## RESULTS

During the study period there were 3400 deliveries, of which 2158 patients delivered vaginally, and 1242 patients had a caesarean section data of hysterotomy (7) and obstetrical hysterectomy (20) were included in the caesarean section, as it was the maternal outcome and could not be categorized separately. This makes the CS rate of our hospital 36.5%. There were 482 (38.8%) primigravida and 760 (61.2%) multigravidas. CS rate was calculated in each group to find its contribution to the overall CS rate as shown in Table-I. Group-5 showed the highest rate of caesarean section 56% (695). Second to it was foetal distress 14% (178). 13.5% were Group-2 nulliparous with inductions or a caesarean section done before labour. Group-1, 11, 6, 9 contributed 5.8% (72), 36 (2.9%), 2.3 (2.3%) respectively. Groups-3, 4, 10 and 8 showed the minimum rate collectively of 4.3%. Group-6 and 7 were breech deliveries (39) delivered via caesarean section, while 89 breeches delivered vaginally. Foetuses of less than 2.5 kg were 178 (14.3%), between 2.5 to 4kg were 1032 (83.1%) and greater than 4kg were 32 (2.6%) as shown in Table-II. Frequencies of age groups, gestational age, procedure done and weight of outcome were compared according to gravida parity with Χ^2^-test and showed significant difference in all parameters except that in weight of outcome.

**Table-I:** Frequencies of Indications for Caesarean Sections as per Robson’s Criteria.

Group	Indications	N	%
1	Nulliparous single cephalic >37weeks spontaneous labour	72	5.8
2	Nulliparous single cephalic >37 weeks induction or caesarean section before labour	168	13.5
3	Multiparous except previous caesarean sections single cephalic >37 weeks spontaneous labour	1	.1
4	Multiparous except previous caesarean sections single cephalic >37 weeks induction or caesarean before labour	12	1.0
5	Previous caesarean section single cephalic >37 weeks	695	56.0
6	All nulliparous breech	29	2.3
7	All multiparous breech including previous caesarean sections	8	.6
8	All multiple pregnancies including previous caesarean sections	16	1.3
9	All abnormal lies including previous caesarean sections	26	2.1
10	All single cephalic >36 weeks including previous caesarean sections	1	.1
11	Placenta Previa and Placenta Accreta Spectrum	36	2.9
12	Foetal distress	178	14.3

Total		1242	100.0

**Table-II:** Cross-tabulation of Age Group, Gestational Age, Procedure Done & Weight of Outcome with Gravida Parity with χ^2^ Test.

		Gravida Parity				

		Primigravida		Multigravida		P Value*

		n	%	n	%	
Age Groups	< 20 Years	280	94.3%	17	5.7%	<.001
	20-35 Years	200	23.9%	637	76.1%	
	> 35 Years	2	1.9%	106	98.1%	
Gestational Age	36-40 Weeks	237	28.9%	583	71.1%	<.001
	< 36 Weeks	43	27.0%	116	73.0%	
	> 40 Weeks	202	76.8%	61	23.2%	
Procedure Done	ELLSCS	4	80.0%	1	20.0%	.010
	EMLSCS	474	39.2%	736	60.8%	
	Hysterotomy	1	14.3%	6	85.7%	
	Laparotomy	3	15.0%	17	85.0%	
Neonatal Weight	< 2.5 kg	63	35.4%	115	64.6%	.406
	2.5-4.0 kg	404	39.1%	628	60.9%	
	> 4 kg	15	46.9%	17	53.1%	

* Significance = ≤.05.

## DISCUSSION

The Caesarean Section in Obstetrics has brought an unexpected change, leading eventually to an increase in maternal morbidity and mortality. Despite the benefits, the associated complication such as infection, bleeding, anaesthetic complication, and the well-known complication of Placenta Accreta Spectrum cannot be neglected.^6^ The future Obstetrics is being a challenge, as going through the stress of saving two lives without any morbity, is not dealt easily. Mostly complicated cases are referred to tertiary care centres as very few private hospitals find the courage to deal with it. The socioeconomic burden on a poor person is far too much resulting in losing lives in a resource limited setting. The risks are far too high when there are financial concerns.^6^,^7^ In order to optimize and judge the right decision of carrying out a caesarean section Robson’s ten group classification system was developed.^5^

JPMC, being a tertiary care centre, receives referrals and caters to all booked and un-booked patients. Majority of patients are critically ill, contributing to the high CS rate. Our CS rate was 36.5 which is similar to Holy Family Hospital 37%. It is much lower than Fauji Foundation Hospital (49%),^7^ Pak Emirates Military hospital (54%)^8^ and Combined Military Hospital, Rawalpindi (56%).^9^

As in other studies, Group-5 included the maximum number of patients. Vogel et al came to a conclusion that previous caesarean section is increasing along with the rise in CSs as observed in this study.^10^ Our findings were similar to the study at Oman (58.2)^11^ and India (94.49).^12^ Our CS rate was lower than the study at PIMS Hospital Islamabad (80.3%).^13^ Our CS rate of Group-5 was much lower than studies carried out at Peru (81%)^14^ and Ghana (71%).^15^ The variation in CS rates in different hospitals must be due to different approaches, expertise available, common risk factor, facilities available and interpretation of findings. Our hospital is reluctant to induce patients with scar, although patients with favourable findings after caesarean are delivered vaginally. This was the main reason of Group-5 had the highest percentage. Only 32 patients delivering vaginally, shows that emphasis should be made on safe (VBAC).

Foetal distress was the second group after Group-5, leading to caesarean deliveries being the only tool to assess foetal distress, results in unnecessary caesarean births. Our findings (14.3%) are similar to a study conducted at PEMH Rawalpindi (13.4%).^8^

On the contrary, Group-2 (12.5%) ranking third in the study showed a lower percentage than the study at PEMH Rawalpindi (18.1%)^8^ and 28.44% in India^12^ The study findings in India and our study are similar making Group-5 and Group-2 being the main cause of the caesareans. This may be due to the large referrals of high risks patients received in both hospitals. The fact that 38.8% were Primigravida was another reason of Group-2 being third on the list. The CS rate in Group-2 was quiet low in our study. Robson suggested 35% as standard for Group-2. This shows that our hospital is quite vigilant while performing a CS on primigravida. Our protocol and aim at JPMC is to put maximum effort on primigravida as, maternal morbidity is minimum in primigravida, of course keeping risks and benefits in mind. Inductions are planned carefully to prevent unnecessary CS.

Group-1 contributed 4rth (5.8%) in the study. This seems a reasonable figure. These figures are similar to a study of Abdul Rahman et al (4.6%).^16^ Our findings are lower than the study of Abu Bekar (26.7%).^6^ The study at Rawalpindi PEMH^17^ shows a lower figure than our study (2.82%). PAS was categorized in separate group as we receive a lot of patients, in this group. Group-11 contributed 2.9% to the overall CS rate. These figures were 0.33% at PEMH Rawalpindi.^17^

Group-6 (2.3%), Group-7 (0.6%) and Group-9 (2.1%) were non cephalic group. External cephalic version is a standard practice at JPMC due to which the percentages are quite low. For Group-9, CS is essential, so our data are aligned with other studies.^1^ Group-8 (1.3%) had a low ratio as patients with twin pregnancy were fewer. Group-3, 4 and 10 had 1%. Group-3 and 4 belongs to multiparous women without scar, with or without induction. The data shows the positive attitudes of doctors at JPMC.

### Limitations

It has the limitations of being a retrospective study.

## CONCLUSIONS

Modified Robson’s criteria is a powerful and essential tool to assess CS rate, of any setup whether it’s at global level or at institutional level or on a small scale. It helps to keep a check on the indications of CS as it is easy and understandable. VBAC should be encouraged to limit Group-5 being the main cause of rising caesareans. Limiting CS sections in primigravida and low risk pregnancy is the only way to reduce CS. Seniors should assess the diagnosis of foetal distress, as mostly its misdiagnosed.

### Authors’ Contribution:

**EM:** Conception & design, initial drafting of manuscript.

**SK:** Analysis and interpretation of the data & drafting of the manuscript.

**SF:** Drafting of manuscript.

**BFZ:** Critical revision of the article for important intellectual content & final approval of the article.

All authors have approved the final version of the manuscript and are responsible for data integrity.
